# Reply to “Serum neurofilament light chain and small fiber neuropathy: Every cloud has a silver lining” by D. Plantone and G. Primiano

**DOI:** 10.1111/ene.16245

**Published:** 2024-02-20

**Authors:** Panoraia Baka, Frank Birklein, Stefan Bittner, Claudia Sommer, all coauthors

**Affiliations:** ^1^ Department of Neurology University Medical Center of the Johannes Gutenberg University Mainz Mainz Germany; ^2^ Department of Neurology University Hospital of Würzburg Würzburg Germany

To the Editor,

We wish to thank Drs Plantone and Primiano for their insightful comments on our study. We fully agree that having a marker, that is specific for peripheral nerve damage would be appealing. However, we do not think that the claim for neurofilament light chain (NfL) being a marker of just central nervous system damage is justified. Numerous studies on peripheral nerve disease, most notably Guillain–Barré syndrome (GBS), and similar acute neuropathies, have shown that nerve‐root and blood‐nerve barrier disruption are important determinators of serum NfL levels [1, 2]. The negative correlation with sural sensory nerve action potential amplitude, even in our cohort with small fiber neuropathy (SFN), is indeed intriguing, as it has been shown in other neuropathies [2].

Furthermore, while the first study on peripherin is encouraging [3], there are still some questions to answer: Can the results be confirmed by independent studies? Will the assay be robust and can the step to broad availability be taken? What do normative control cohorts look like and which confounders need to be taken into account? Is peripherin really peripheral nervous system‐specific? There are some doubts about this, as a few multiple sclerosis patients and healthy subjects also had increased levels. When these questions are answered, we will have entered the next level of monitoring peripheral nerve damage. Currently, we think that assessing both NfL and, for example, peripherin, in parallel in larger cohorts would be interesting. As both assays measure very low protein levels, unknown factors related to the technical feasibility of the assays and potential confounders may impact the results. It would also be very interesting to assess the dynamics of NfL and peripherin blood levels during and after nerve damage: How high is the increase and how long does it last? We and others have shown that NfL levels in peripheral neuropathies are higher in the acute stage and diminish over time, even if axonal damage is still present [1, 4]. Studies of different peripheral nerve diseases (e.g., GBS, chronic inflammatory demyelinating polyneuropathy, SFN, trauma) will be needed to obtain a clear picture.

We also agree that several factors, such as age, chronic kidney disease and body mass index (BMI), impact the measurement of NfL concentrations and limit the possibility to establish fixed cut‐off levels of absolute NfL concentrations. Recent efforts to establish large reference databases for adults and children all found that age was the most important confounder. Compared to age, the role of BMI seems to be small and is relevant mainly in cohorts with obese patients (BMI >30 kg/m^2^). Therefore, BMI was included only in the reference cited by Drs Plantone and Primiano, but not in others because of its small effect on NfL concentrations [5]. We acknowledge that the lack of BMI in our study is a potential limitation, but we do not believe that this has skewed our findings because none of our subjects was obese.

## AUTHOR CONTRIBUTIONS

**P. Baka:** Conceptualization; writing – original draft; writing – review and editing. **F. Birklein:** Conceptualization; writing – original draft; writing – review and editing; supervision. **S. Bittner:** Conceptualization; writing – original draft; writing – review and editing; supervision. **C. Sommer:** Conceptualization; writing – original draft; writing – review and editing; supervision.

## Data Availability

The data that support the findings of this study are available on request from the corresponding author. The data are not publicly available due to privacy or ethical restrictions.
